# A 3D CFD model of the interstitial fluid pressure and drug distribution in heterogeneous tumor nodules during intraperitoneal chemotherapy

**DOI:** 10.1080/10717544.2019.1588423

**Published:** 2019-03-31

**Authors:** Margo Steuperaert, Charlotte Debbaut, Charlotte Carlier, Olivier De Wever, Benedicte Descamps, Christian Vanhove, Wim Ceelen, Patrick Segers

**Affiliations:** a Biofluid, Tissue and Solid Mechanics for Medical Applications (bioMMeda), Department of Electronics and Information Systems, Ghent University, Ghent, Belgium;; b Departement of GI Surgery and Cancer Research Institute Ghent (CRIG), Ghent University, Ghent, Belgium;; c Department of Human Structure and Repair, Ghent University, Ghent, Belgium;; d Infinity (iMinds-IBiTech-MEDISIP), Department of Electronics and Information Systems, Ghent University, Ghent, Belgium

**Keywords:** Drug transport, intraperitoneal chemotherapy, interstitial fluid pressure, DCE-MRI, computational fluid dynamics

## Abstract

Although intraperitoneal chemotherapy (IPC) has evolved into an established treatment modality for patients with peritoneal metastasis (PM), drug penetration into tumor nodules remains limited. Drug transport during IPC is a complex process that depends on a large number of different parameters (e.g. drug, dose, tumor size, tumor pressure, tumor vascularization). Mathematical modeling allows for a better understanding of the processes that underlie drug transport and the relative importance of the parameters influencing it. In this work, we expanded our previously developed 3D Computational Fluid Dynamics (CFD) model of the drug mass transport in idealized tumor nodules during IP chemotherapy to include realistic tumor geometries and spatially varying vascular properties. DCE-MRI imaging made it possible to distinguish between tumorous tissues, healthy surrounding tissues and necrotic zones based on differences in the vascular properties. We found that the resulting interstitial pressure profiles within tumors were highly dependent on the irregular geometries and different zones. The tumor-specific cisplatin penetration depths ranged from 0.32 mm to 0.50 mm. In this work, we found that the positive relationship between tumor size and IFP does not longer hold in the presence of zones with different vascular properties, while we did observe a positive relationship between the percentage of viable tumor tissue and the maximal IFP. Our findings highlight the importance of incorporating both the irregular tumor geometries and different vascular zones in CFD models of IPC.

## Introduction

Cancers originating from organs in the peritoneal cavity are prone to loco-regional spread in the form of peritoneal metastasis (PM). The prognosis of patients who develop PM is usually poor and quality of life is low due to complications, such as bowel obstructions and ascites. Furthermore, PM cannot be adequately treated by using intravenous (IV) chemotherapy due to the poor blood supply to the peritoneal surfaces and poorly vascularized tumor nodules (Goodman et al., 2016). Dedrick et al. (1978) hypothesized that the peritoneum-plasma barrier offers a unique treatment opportunity for patients with oncological malignancies confined to the peritoneal cavity, introducing intraperitoneal drug delivery as a therapeutic strategy. Despite a strong rationale and promising clinical results (Miyagi et al., 2005), widespread use of the technique is currently hampered by the limited penetration depth of the drugs into the tumor tissue (Los et al., 1990; Royer et al., 2012; Ansaloni et al., 2015). It is, therefore, crucial to gain a better understanding of the processes that underlie the drug transport and the relative importance of the parameters influencing it.

Intraperitoneal drug delivery encompasses a complex transport process that depends on a large number of different parameters. The final drug distribution in the tumor tissue is influenced by therapy-related factors such as dose, temperature, (volume of) carrier fluid, intra-abdominal pressure, the potential use of vaso-active agents or surfactant and duration. It is also heavily dependent on factors related to the drug itself like molecular weight, ionic charge, membrane binding, solubility, diffusivity and the on properties of the tumor tissue (e.g. permeability, vascularity, interstitial fluid pressure (IFP), cell density, extracellular matrix (ECM) composition, …) (Steuperaert et al., 2017). Due to its relatively low cost and versatility, mathematical modeling has become an important research tool to better understand and optimize drug delivery. Most existing models of chemotherapeutic drug delivery, however, have been created for systemic drug delivery (Kim et al., 2013), while only a limited number of models focuses on intraperitoneal chemotherapy (Steuperaert et al., 2017). Historically, intraperitoneal drug transport has often been described using a compartmental model (El-Kareh & Secomb, 2003; Miyagi et al., 2005; Shah et al., 2009; Colin et al., 2014; Goodman et al., 2016), in which drug concentrations are typically averaged over the entire tumor. More recent works also take into account spatial variations in drug concentrations on a tissue level (Flessner et al., 1985; El-Kareh & Secomb, 2004; Flessner, 2005; Stachowska-Pietka et al., 2012; Au et al., 2014; Steuperaert et al., 2017) and even on the single-cell level (Winner et al., 2016).

The use of DCE-MRI to gain information about physiological tissue characteristics has been previously applied to the field of oncology. Pishko et al. (2011) created spatially-varying porosity and vascular permeability maps from the two-compartment analysis of DCE-MRI data using a rescaled AIF from literature. They incorporated these maps in a 3D computational fluid dynamics (CFD) porous media model to predict interstitial fluid pressure and velocities (IFP and IFV respectively) as well as tracer transport in mice sarcomas. Using the same mathematical model, DCE-MRI dataset and post-processing techniques, Magdoom et al. (2012) used a voxelized modeling methodology to eliminate the time-intensive tumor segmentation step. Zhao et al. (2007) similarly used DCE-MRI to generate normalized spatial variation maps of vascular permeability to calculate IFV, IFP, and tracer transport within a solid murine tumor. The tracer concentration in the plasma was assumed to be proportional to the relative change in signal intensity and AIF functions were taken from literature. The effect of heterogeneous microvessel density extracted from DCE-MRI on drug concentrations in the extra- and intra-cellular space studied by Zhan et al. (2014). Using a 2 D liver tumor geometry, the tracer concentration in the plasma was assumed to be proportional to the relative change in signal intensity. Bhandari et al. (2017) used DCE-MRI data and patient-specific AIFs to determine kinetic perfusion parameters in human brain tumors and predict IFP, IFV, and tracer transport.

Previously, we developed a 3D computational fluid dynamics (CFD) model that accounts for the diffusive, convective, and reactive drug transport during intraperitoneal chemotherapy (Steuperaert et al., 2017). We demonstrated the important influence of both tumor nodule geometry and interstitial fluid pressure (IFP) on the penetration depth of cytotoxic drugs in idealized geometries. In this paper, we extend our previous work and present a workflow to implement both realistic geometries and IFP profiles in our existing 3D CFD model. The input for the computational model was obtained from experiments in a murine cancer model. The IFP data was obtained both by direct measurements using a pressure tip catheter and estimated from dynamic contrast-enhanced magnetic resonance images (DCE-MRI). The modeled geometries were all mouse-specific based on anatomical MRI images.

## Material and methods

**Figure 1.**
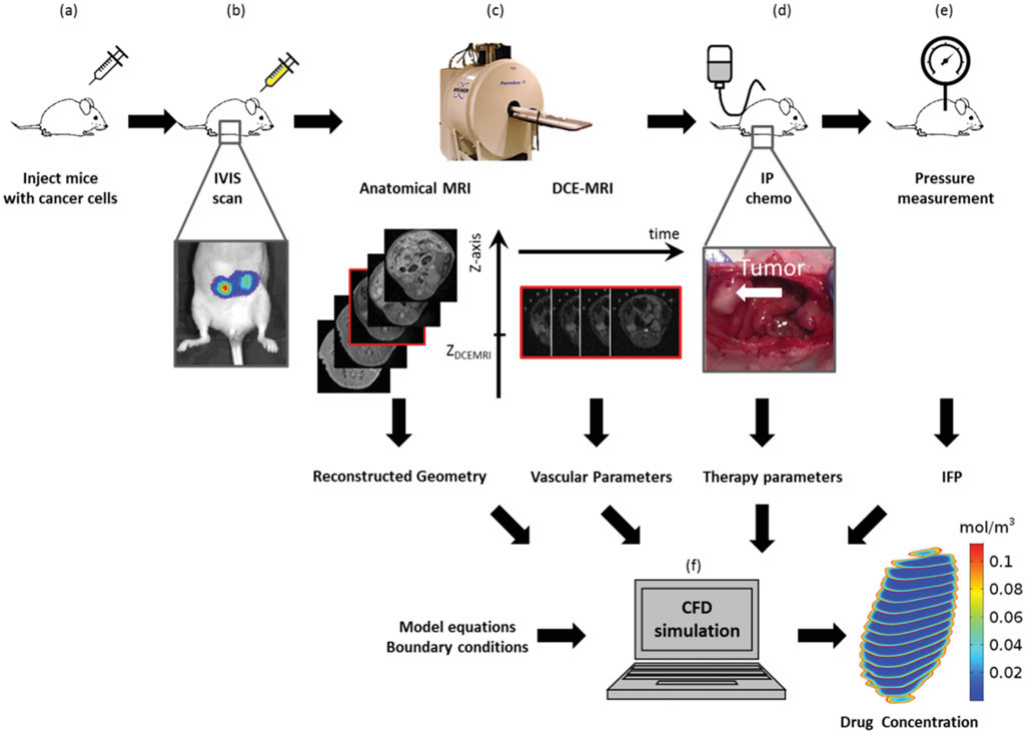
Schematic illustration of the workflow described in this work. (a) Subperitoneal injection of SKOV3-Luc-IP1 cells suspended in Matrigel in female athymic, nude-foxn1nu mice by a growth phase. (b) *In vivo in situ* (IVIS) scan was done 14 days post-injection to assess tumor growth. (c) Scanning protocol as described in the section *MRI protocol* consisting of an anatomical scan to segment the tumor geometries, a FLASH sequence to obtain native relaxation times of the tissues and a DCE-MRI from which vascular parameters of the tumor tissues were derived. The anatomical MRI slice with a red circumference has the same applicate as the DCE-MRI plane. (d) Mice positive for tumor growth underwent 30 min of intraperitoneal chemotherapy (IPC) with cisplatin. The therapy parameters (dose, duration) that were used in the experiment were used in the model setup as boundary conditions. (e) Immediately after IPC, the IFP was measured using a pressure tip catheter. The resulting pressures were used to validate the simulated tumor pressures in a later stage. (f) The equations for both the IFP build-up using the vascular parameters derived from the DCE-MRI data and the drug mass transport were implemented in COMSOL and solved using the same initial and boundary conditions as the experimental setup. The resulting pressure and drug distributions are reported in the ‘results’ section.

A mouse PM model was used from which tumor geometries could be obtained and IFP could be measured invasively. As described in more detail below, we performed an imaging protocol to monitor tumor growth and to estimate tumor IFP values from DCE-MRI parameters. Pressure and concentration distributions were then calculated using the estimated, spatially varying transport parameters obtained based on the DCE-MRI images. Figure 1 shows a schematic illustration of the workflow.

All animal experiments were approved by the local Ethics Committee on Animal Experiments of the Faculty of Medicine, Ghent University, and were performed according to Belgian and European legislation on animal welfare (Directive 2010/63/EU). Animals were group housed and kept under environmentally controlled conditions (12 h light/dark cycles, 20–23 °C, 40–60% relative humidity) with food and water *ad libitum*.

### Cell line

Human ovarian cancer cells (SKOV3-Luc-IP1) (De Vlieghere et al., 2016) were cultured in Dulbecco's Modified Eagle’s Medium (Life technologies/ThermoFisher, Ghent, Belgium) and supplemented with 2% penicillin/streptomycin + 0.005% fungizone (Bristol-Myers-Squib B.V., Utrecht, The Netherlands) and 10% fetal calf serum (Sigma-Aldrich, Diegem, Belgium).

### Mouse model

Eight weeks old, female athymic, *nude-foxn1nu* mice (21 g average body weight, ENVIGO, NM horst, the Netherlands) were monitored for general health during one week before the start of the study. After conditioning, 12 mice underwent a midline laparotomy under general anesthesia with Sevoflurane (Baxter, Deerfield, USA) after which they were bilaterally injected via the subperitoneal route with 5.0 × 10^5^ SKOV3- Luc-IP1 cells suspended in 50 μl of Matrigel® (Life Sciences, Antwerp, Belgium) to facilitate the growth of peritoneal tumor nodules (Figure 1(a)). All mice were given subcutaneous pain relief (Ketoprofen, 150 µl) immediately after the procedure. Their weight and general wellbeing were monitored during recovery.

To assess the success rate of tumor induction and monitor tumor growth, a bioluminescence scan (IVIS Lumina II, Perkin Elmer) was acquired 14 days post-injection (Figure 1(b)). Each animal was injected intraperitoneally with Luciferine (D-Luciferin, PerkinElmer, Waltham, USA) using a dose of 150 mg/kg body weight. During the first scan, a calibration series was performed to assess the time after injection at which the signal was maximal. For subsequent scans, the same waiting period was maintained.

### MRI protocol

MRI scanning (Figure 1(c)) was performed using a 7 T MR system (PharmaScan 70/16, Bruker, Ettlingen, Germany) with a mouse body volume coil. During the scanning protocol, all mice were anesthetized with isoflurane (5% induction, 1.5% maintenance, 0.3 L/min) and their body temperature was maintained using a heating blanket. An anatomical scan was obtained using a T1-weighted sequence (RARE) with the following settings: repetition time (TR) 1455 ms, echo time (TE) 9.0184 ms, flip angle (FA) 180°, in-plane resolution 120 μm, slice thickness 600 μm, and an acquisition of 30 contiguous transverse slices. Using this anatomical scan, a single slice was chosen in which both tumor nodules were visible. Native relaxation times were calculated in this slice from a FLASH sequence with four different TR values, i.e. 100 ms, 502 ms, 1184 ms, and 5000 ms. Other scanning parameters were: TE 100 ms, FA 180°, in-plane resolution 268 μm. The DCE-MRI series was acquired for the chosen slice using a FLASH sequence with TR 12 ms, TE 3.4 ms, FA 25°, in-plane resolution 268 μm, 550 repetitions, and temporal resolution 1.344 s, resulting in a total acquisition time of 12 min 19 s. After 60 s of baseline signal measurements, a bolus of 0.2 mmol/kg Gd-DOTA (Dotarem™, Guerbet, Paris, France) was injected through a tail vein catheter. Animals that did not show tumor growth were excluded from the rest of this study.

### Intraperitoneal chemotherapy

Three weeks after tumor inoculation, all mice positive for tumor growth were put under general anesthesia and underwent 30 min of intraperitoneal chemotherapy (IPC) (Figure 1(d)) with cisplatin (Hospira Benelux BVBA, Antwerp, Belgium). The administered cisplatin dose was calculated as 1/100th of the clinically used dose relative to the body surface area (BSA) resulting in used values of 0.7 mg cisplatin/mouse (concentration in solution of 113 µmol/l). The cisplatin solution circulated in a circuit consisting of an inlet and outlet probe fitted with temperature sensors (TM 9604; Ellab A/S, Hilleroed, Denmark), a connecting tubing that passed through a 520 U process pump (Watson-Marlow NV, Zwijnaarde, Belgium) and a M3 LAUDA heat exchanger (LAUDA-Brinkmann, New Jersey, USA) (Gremonprez et al., 2015). All procedures were performed on a heating pad and the chemotherapeutic solution temperature was kept at 37–38 °C (normothermic conditions).

### Interstitial fluid pressure measurement

Immediately after IPC, the chemotherapeutic solution was drained from the abdominal cavity, after which the peritoneal surfaces were pat dried. A SPR-320 pressure tip catheter connected to a PCU-2000 pressure control unit and PowerLab 35 Series data acquisition system (Millar, Houston, USA) was manually inserted in the center of each tumor and held there until a stable pressure output signal was measured (Gremonprez et al., 2015).

### Data processing, fitting, and interpolation

During DCE-MRI scanning, a series of MRI scans are acquired in rapid succession following the intravenous injection of the paramagnetic contrast agent. The underlying principle of the technique relies on the fact that as the contrast agent disperses through the tissue, it changes the MR signal intensity of the tissue depending on its local concentration.

Following Zhu et al. (2000), T2* relaxation was neglected to compute the contrast profiles due to the use of short TR’s and TE’s (rendering the attenuation of signal due to the terms related to T2* contribution (e^−TE/T2^*) negligible for low contrast concentrations). We thus applied Equation (1a) for the contrast concentration *c:*

(1a) $$c=\frac{1}{r_{1}TR}l\mathrm{og}\left[E_{10}\frac{1+\text{cos}(\alpha )(\mathrm{RIE}(E_{10}-1)-1)}{\mathrm{RIE}\left(E_{10}-1\right)+E_{10}(1-\text{cos}\left(\alpha \right))}\right]$$

with

(1b) $$E_{10}=\exp(-\frac{\mathrm{TR}}{T_{10}})$$

The relative intensity enhancement (RIE) was calculated as $\mathrm{RIE}=\frac{S-S_{0}}{S_{0}}$ with $S_{0}$ the baseline signal calculated from 60 s of measurement prior to contrast injection, *S* the signal at that timepoint and $r_{1}$ is the relaxivity of Dotarem (3,5 mM^−1^s^−1^ (http://www.ajnr.org/content/ajnr/suppl/2014/05/22/ajnr.A3917.DC1/3917_tables.pdf)) in the interstitial fluid, $T_{10}$ the native relaxation time calculated from the four images with different TR, and α is the flip angle. Equation (1a) is solved for each pixel of the tumorous region of interest (ROI) and for each timepoint after contrast injection. By tracking the concentration values of each pixel in function of time, *c(t)* profiles can be calculated.

In addition to calculating the concentration profiles of the contrast agent from the equations above, we can describe the transient concentration of the Gd-DOTA (*c_GD_*) tracer in the region of interest by a two-compartment model (Zhao et al., 2007), with the two compartments being the interstitial space and the blood plasma:

(2) $$\varphi \cdot \frac{dc_{\mathrm{GD}}}{\mathrm{dt}}=P\frac{S}{V}\left(c_{\mathrm{AIF}}-c_{\mathrm{GD}}\right)+\frac{J_{v}}{V}(1-\sigma )c_{\mathrm{AIF}}$$

in which $c_{\mathrm{GD}}$ and $c_{\mathrm{AIF}}$ are the concentrations of the tracer in the interstitial space and the plasma, respectively. $c_{\mathrm{AIF}}$ is also known as the arterial input function (AIF). φ is the interstitial fluid volume fraction, P is the permeability coefficient of the vasculature for the tracer, $\frac{S}{V}$ is the surface to volume ratio of the vasculature, σ the osmotic reflection coefficient for the tracer, and $\frac{J_{v}}{V}$ the plasma filtration rate per unit volume. In order to obtain a good estimate for the parameters φ,$P\frac{S}{V}$ and $\frac{J_{v}}{V},$ it is crucial to provide an AIF that is as accurate as possible. To extract a mouse-specific AIF from the DCE-MRI dataset, a high temporal resolution is needed throughout the series. This in turn, limits the spatial resolution that can be achieved in the DCE-MRI plane. To obtain the AIF, Equation (1) was again used to calculate the contrast concentration on candidate AIF pixels that were identified on the anatomical image. The contrast concentration calculated in these pixels represents the blood concentration of contrast ($c_{b}$) so to obtain the plasma concentration of contrast ($c_{\mathrm{AIF}}$), an additional scaling had to be performed, taking into account the hematocrit value (Hct) of the mouse (Equation (3)) (Tofts & Parker, 2013).

(3) $$c_{\mathrm{AIF}}=\;\frac{c_{b}}{1-\mathrm{Hct}}\;$$

The resulting plasma concentration curve was then filtered and fitted to a bi-exponential curve that was further used in its analytical form as the AIF. Using the corresponding mouse-specific AIF for each tumor ROI, the solution to this first-order differential equation (*c_GD_*, Equation (2)) can be fitted on a voxel to voxel basis to the contrast profiles that were calculated from the DCE-MRI (Equation (1a)) series to obtain estimates for φ,$P\frac{S}{V}$ and $\frac{J_{v}}{V}.$

To implement spatially varying vascular parameters in our model we used an approach previously described by Zhao et al. (2007) in which the $\frac{J_{v}}{V}$ values are rescaled to the product of the vascular hydraulic conductivity and surface-to-volume ratio of the microvasculature $L_{p}\frac{S}{V}.$ In order to do so, all $\frac{J_{v}}{V}$ values were first normalized with respect to the average value$\;({\frac{J_{v}}{V})}_{\mathrm{mean}}$ of all fitted pixels in the ROI and then scaled by multiplying the normalized values with the product of the baseline values typically used for hydraulic conductivity and vascular surface to volume ratio in literature (Gremonprez et al., 2015) ($L_{p,o}=2.1\times 10^{-11}\frac{m}{\mathrm{Pa}\cdot s};\;{\left(\frac{S}{V}\right)}_{0}=\;2.00\;\times 10^{4}\;m^{-1})\;$(Equation (4)).

(4) $${\left(\frac{L_{p}S}{V}\right)}_{i,j}=L_{p,0}\cdot {\left(\frac{S}{V}\right)}_{0}\cdot \frac{{\left(\frac{J_{v}}{V}\right)}_{i,j}}{{\left(\frac{J_{v}}{V}\right)}_{\mathrm{avg}}}$$

The extrapolated $\frac{L_{P}S}{V}$ values were then used as input for the Starling term in our model for IF flow (see further).

The need for a high temporal resolution (to extract the AIF from the data) and the available MRI hardware, limited us to a 2-dimensional DCE-MRI series. To provide 3D spatially varying parameters, extrapolation of the available data was needed. Upon inspection of the estimated resulting parameter maps, two distinctively different regions could be detected in each tumor. In tumors 1 and 2, there were interior zones with pixels that could not be fit by Equation (2), whereas the surrounding tissue was fit well by the same equation. In tumor 3 on the other hand, the majority of interior pixels was well fit but two zones for which the $\frac{L_{P}S}{V}$ parameter yielded very different results, could be distinguished. These different regions were then related back to the anatomical scans (T1 weighed scans) and traced in each slice of the anatomical scan of the tumor geometry thereby effectively segmenting a second 3D zone within the original tumor. The mean $\frac{L_{P}S}{V}$ value averaged over all fitted pixels in the sub-ROI was then assigned to each of the different regions. Implications of this extrapolation will be discussed in the discussion section.

### Computational model

For this work, three different tumors were selected based on their respective. The tumor geometries were segmented using Mimics (Materialise, Leuven, Belgium) and the resulting geometries were smoothed and meshed in 3-Matic (Materialise, Leuven, Belgium) before they were imported as stl-files in COMSOL Multiphysics (COMSOL, Inc., Burlington, USA). A similar procedure was followed to obtain the boundary of any additional internal zones that were present in the tumors. Volume meshes where created in COMSOL Multiphysics using the same element size for each geometry (1.7 × 10^−4 ^mm³) based on the mesh independence study of the model, resulting in the mesh sizes listed in Table 1. The equations for both the IFP build-up and the mass transport that were implemented in COMSOL were previously described by our group (Steuperaert et al., 2017) and summarized below. In a rigid porous medium like the tumor interstitium, the momentum equation can be reduced to Darcy’s Law (Bird et al., 2007):

(5) $$u=-K\nabla P_{i}$$

**Table 1a.** Geometrical properties of the three segmented geometries.

	Tumor 1	Tumor 2	Tumor 3
Geometrical properties			
Tumor location	Right	Left	Right
Tumor Size	8 × 11 mm	6 × 8 mm	4 × 6 mm
Reconstructed tumor volume	288 mm³	121 mm³	45 mm³
Reconstructed interior volume	69 mm³	9 mm³	8 mm³
Mesh size (number of volume elements)	1693526	715057	421569
#pixels in DCE-MRI ROI	329	270	181

**Table 1b.** Summary of pressure and penetration depth results.

	Tumor 1			Tumor 2		Tumor 3	
	SA1	SA2	LA	SA	LA	SA	LA
Pressure and concentration results							
Pmeas(Pa)	2067			2890		2533	
Pmax (Pa)	1385	1429	1411	1523	1525	1428	1468
LP50 (mm)	L: 0.0437	L: 0.0407	L: 0.0290	L: 0.0565	L: 0.0365	L: 0.0819	L: 0.0727
	R: 0.0428	R: 0.0351	R: 0.0401	R: 0.0519	R: 0.0378	R: 0.0708	R: 0.0603
APD (mm)	L: 0.361	L: 0.320	L: 0.412	L: 0.370	L: 0.422	L: 0.371	L: 0.495
	R: 0.331	R: 0.327	R: 0.404	R: 0.328	R: 0.281	R: 0.394	R: 0.463
PD% (%)	L: 5.63%	L: 5.50%	L: 5.33%	L: 7.96%	L: 4.96%	L: 10.3%	L: 7.92%
	R: 5.16%	R: 5.68%	R: 5.23%	R: 6.85%	R: 3.30%	R: 10.9%	R: 7.41%
Pvol% (%)	28.04%			31.32%		43.42%	

where u represents the interstitial fluid velocity vector (in m/s); K the conductivity of the tissue for interstitial fluid (3.1 × 10^−14^ m^2^/Pa s (Baxter & Jain, 1989)), $P_{i}$ the interstitial fluid pressure (Pa) and ∇ the gradient operator.

The steady-state continuity equation for the incompressible interstitial fluid flow in normal tissue is given by (Bird et al., 2007):

(6) $$\nabla \cdot u=F_{v}-F_{l}$$

were ∇· represents the divergence operator; $F_{v}$ the fluid gain from the blood (s^−1^) and $F_{l}\;$a lymphatic drainage term for interstitial fluid (s^−1^). Since there is a known lack of functional lymphatics in solid tumors, $F_{l}=0.$ The constitutive relation for $F_{v}$ is based on Starling’s hypothesis (Baxter & Jain, 1989) (Equation (7)):

(7) $$F_{v}=\frac{L_{p}S}{V}\left(P_{v}-P_{i}-c\left(\pi_{v}-\pi_{i}\right)\right)\;$$

with *L_p_* the hydraulic conductivity of the vasculature (m/Pa·s), *S/V* the surface to volume ratio of the vasculature (m^−1^), *P_v_* the vascular pressure (Pa), *P_i_* the interstitial fluid pressure (Pa), *c* the non-dimensional osmotic reflection coefficient, π*_v_* the vascular osmotic pressure (Pa) and π*_i_* the interstitial osmotic pressure (Pa). IFP profiles were calculated by solving the steady state form of the momentum and continuity equations. When more than one zone was present in the tumor, the source and sink terms (i.e. Starling source) were adapted in each zone to include the corresponding estimated *L_p_S/V* value as discussed in the ‘data processing’ section.

Mass conservation of the drug is given by Equation (8) (Bird et al., 2007):

(8) $$\frac{\partial C_{\mathrm{drug}}}{\partial t}=D\nabla^{2}C_{\mathrm{drug}}-\nabla \cdot \left(uC_{\mathrm{drug}}\right)-S$$

with $C_{\mathrm{drug}}$ the time-dependent concentration of the drug present in the interstitium (mol/m^3^), D the diffusion coefficient (m^2^/s), $\nabla^{2}$ the Laplacian operator, ∇· the divergence operator and S the sink in drug concentration (mol/m^3^). This sink term includes losses due to cellular uptake and resorption by the vascular system (Steuperaert et al., 2017). To calculate the IF flow, the pressure at the outer edge of the tumor is kept constant at 0 Pa and at the interface between two different tumor zones, an interface boundary condition is imposed, implying continuity of all properties. On the edge of the tumor nodule, where the IPC drug is in direct contact with tumor tissue, a fixed drug concentration is maintained (i.e. 0.113 mol/m³). Initial values for pressure and concentration in the domain were set to 0 Pa and 0 mol/m³, respectively. Values for all model parameters mentioned above were taken from our previously published model of the drug distribution in a single tumor nodule during IP chemotherapy (Steuperaert et al., 2017).

A segregated approach was used for solving the continuity equation, momentum transport, and mass conversation of the drug. All three tumor cases were run as transient models with a time resolution of 30 s. As a convergence criterion, a drop of 4 orders of magnitude in the residuals was chosen.

### Reported parameters

By solving the drug transport equation (Equation (8)) using the pressure and velocity field calculated in the previous section, drug concentrations could be determined in the tumor geometries.

For all simulations, pressure and concentration profiles were analyzed along 2 or 3 perpendicular axes in the XY-plane with the same applicate as the one of the DCE-MRI planes. All lengths reported are distances that are normalized with respect to the corresponding length of the axis. The pressure profile along a certain axis is characterized by the maximal pressure (*P_max_*) along this axis and the steepness of the pressure profile in that direction. In this work, the steepness is characterized by the *LP50* value, which we define as the distance starting from the tumor edge after which the pressure reaches 50% of its maximal value. The steeper the pressure profile, the lower the *LP50* value will be.

From the concentration profiles along the axes, the penetration depth is determined. Both absolute penetration depths (APD) and relative penetration depth percentages (PD%) are reported. The APD is defined as the maximal depth along the axis of interest where the drug concentration exceeds the corresponding half maximal inhibitory concentration value of the drug (*IC50*). The PD% represents the percentage of the length of the axis where concentration values exceed the corresponding *IC50* value of the drug used. The inclusion of different zones with varying vascular properties results in non-symmetric profiles along the axes. To highlight this phenomenon, values for the penetration depths are reported with respect to both sides of the axis (L = smallest abscissa; R = largest abscissa).

## Results

### Geometry and segmentation

**Figure 2.**
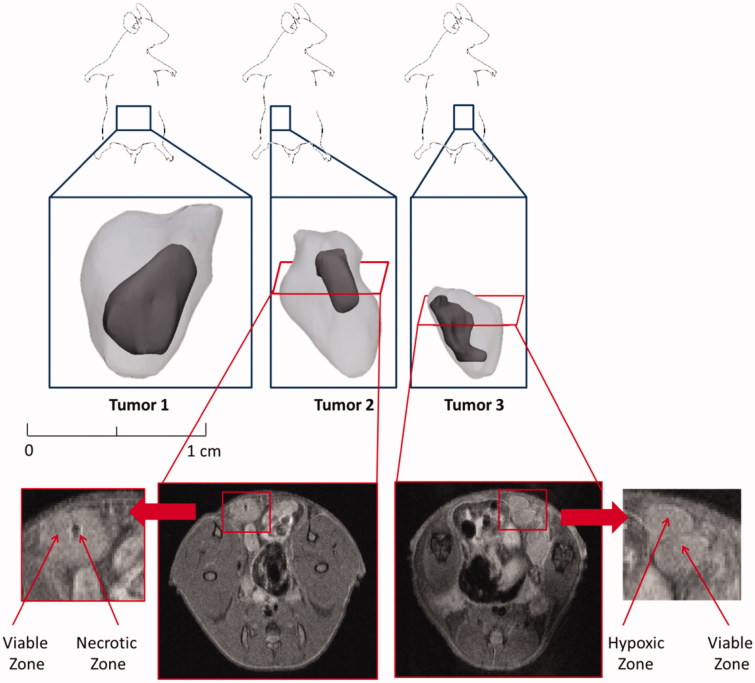
Visualization of the three segmented tumor geometries and their different zones on a common scale. The tumors have reconstructed volume values of 288, 121 and 45 mm³, respectively.

The three selected tumors significantly differed in size with reconstructed tumor volumes ranging from 45 mm³ to 288 mm³. Tumor 1 was the largest with a volume of 288 mm³. The interior zones that can be seen in Figure 2 can be related to the zones with varying vascular properties that were segmented and ranged in volume from 8 mm³ for tumor 3–69 mm³ for tumor 1. The number of pixels in the tumor ROI of the DCE-MRI images varied from 181 to 329. A summary of the geometric properties of the different tumors is presented in Table 1(a).

### Data processing

**Figure 3.**
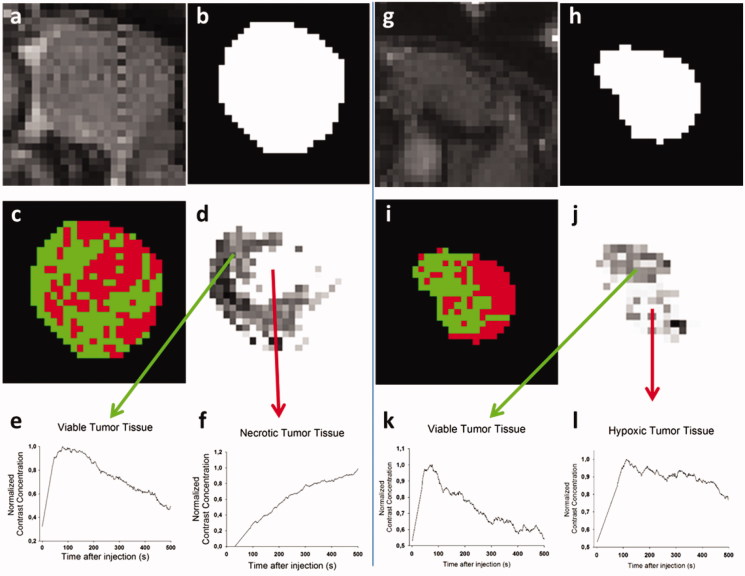
(a) Baseline DCE-MRI image of the tumor 1 ROI. (b) Mask created of tumor 1 ROI based on the baseline image. (c) Visualization of the pixels within the tumor 1 ROI that were adequately fit (*R*^2^ > 0.85) in green and the ones that could not be fit in red. (d) *LpS/V* map of the tumor 1. (e) Representative *c(t)* profile for the pixels in the darker, viable tissue zone of image 3d. (f) Example of a *c(t)* profile for the pixels in the white, necrotic tissue zone of image 3d. Tumor 2 is not included in the figures, but Representative *c(t)* profiles are similar to the ones obtained in tumor 1 with both a viable and necrotic tumor tissue zone, albeit of different size and shape. (g) Baseline image of the tumor 3 ROI. (h) Mask created of tumor 3 ROI based on the baseline image. (i) Visualization of the pixels within the tumor 3 ROI that were adequately fit (*R*^2^ > 0.85) in green and the ones that could not be fit in red. (j) *LpS/V* map of the tumor 3 with two distinctive zones that can be noted. (k) Representative *c(t)* profile for the pixels in the viable tissue zone of image 3j. (l) Example of a *c(t)* profile for the pixels in hypoxic tissue zone of image 3j.

For each of the 3 chosen animals, a suitable AIF could be fitted from the concentration data. Using the animal-specific AIF, the parametric solution for Equation (2) was calculated and subsequently fitted to all pixels within the ROI (ROI tumor 1: Figure 3(b), ROI tumor 2: Figure 3(h)). Upon fitting the pixels of the tumor ROI, we found that certain pixels were not adequately fitted (*R^2^* < 0.85) (red pixels in Figure 3(c,i)). The contrast concentration *c(t)* increased fastest in the viable tissue areas as a result of rapid Gd-DOTA uptake in this well-perfused region, followed by rapid washout (Figure 3(e,k)). In hypoxic areas, however, there is typically a reduced vascularization which is reflected in a delayed Gd-DOTA signal build-up as well as a delayed and prolonged wash-out of signal (Figure 3(l)). In necrotic regions of the tumor, there is no vascularity thought to be present and no washout of the contrast agent could be seen in the signal (http://www.ajnr.org/content/ajnr/suppl/2014/05/22/ajnr.A3917.DC1/3917_tables.pdf) (Figure 3(f)). Unlike pixels in the hypoxic and viable tissue areas, pixels in necrotic areas were not adequately fitted by Equation (2). For the pixels that were adequately fitted, *Jv/V* parameter maps were created and scaled to *LpS/V* maps (Figure 3(d,j)). Upon inspection of the resulting *LpS/V* maps different zones within the tumor nodules could be identified (hypoxic/necrotic/viable tissue). The *LpS/V*-values were then averaged over the different zones and the final values ranged from 0 in the necrotic areas to 3.946 × 10^−7^ (Pa s)^−1^ in the outer zone of tumor 3.

### Pressure measurement

The measured pressures in the three tumors were 15.5 mmHg (2067 Pa), 21.3 mmHg (2890 Pa) and 19 mmHg (2533 Pa) for tumor 1, 2 and 3, respectively. In Table 1(b), the converted pressure values in Pa are summarized.

### Pressure simulation

**Figure 4.**
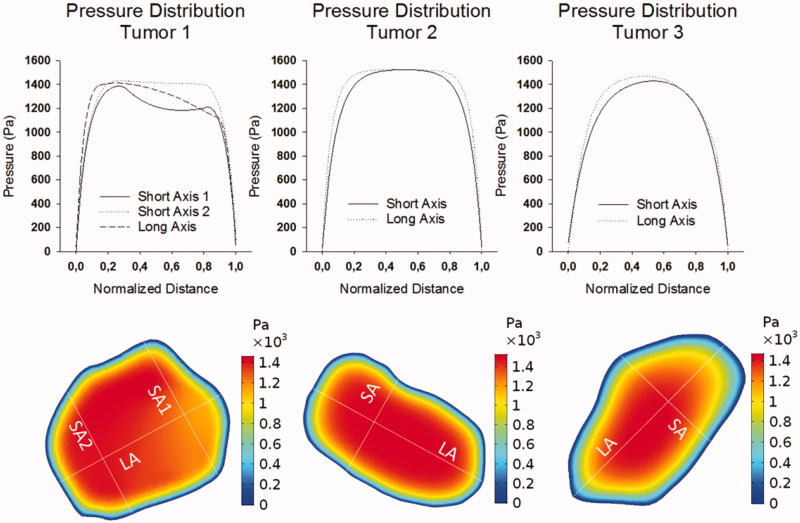
Pressure distributions in the different tumor geometries along the short and long axes (SA and LA, respectively) presented at the bottom; tumor projections are not to scale. Pressures are noted in Pa and the distances are normalized with respect to the total respective length of the axis.

The model can calculate IFP pressures and concentration distributions in all geometries. The maximal IFP (Table 1(b)) reached in our simulations was 1525 Pa (11.44 mmHg), obtained in tumor 2. The shape of the pressure profiles (Figure 4) differed strongly between the different tumor geometries and even along the longer and shorter axes in the same tumor geometry. It is interesting to note that the pressure profile along the short axis 1 of tumor 1 has a local minimum. Another pressure related parameter that showed a directional variability was the *LP50* with a peak value of 0.819 mm for the left side of the long axis of tumor 3 and a minimum of 0.0290 for the left side of the long axis of tumor 1.

### Drug distribution

**Figure 5.**
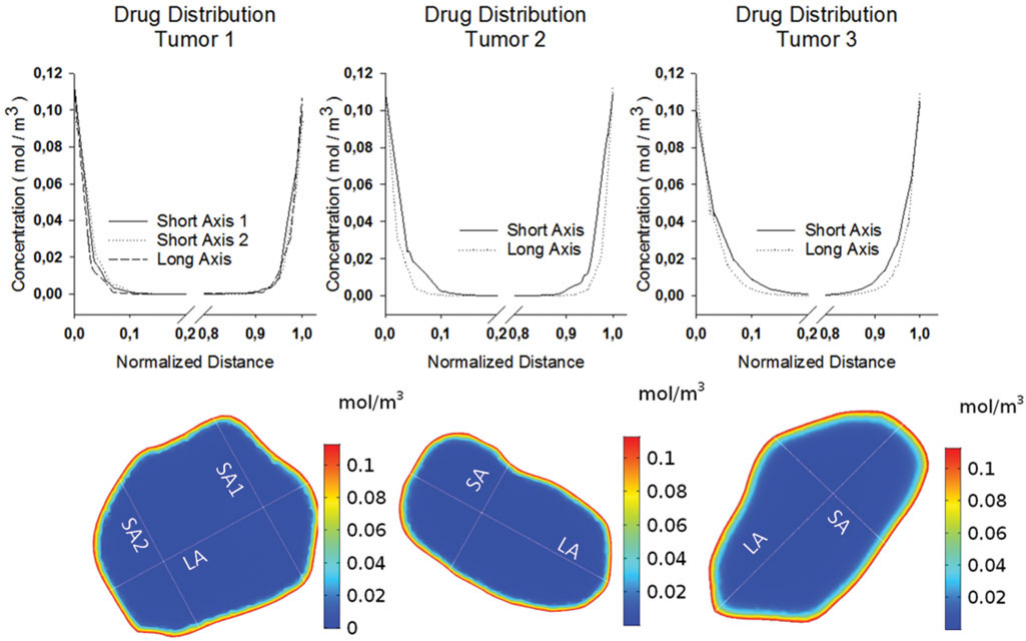
Drug distribution in the different tumor geometries along the axes as shown in the bottom; tumor projections are not to scale. Concentrations are noted in mol/m³ and the distances are normalized with respect to the total respective length of the axis.

Drug penetration was analyzed along the same axes mentioned above and all concentrations were normalized with respect to the initial concentration *c* = 113 µmol/l. Absolute penetration depths ranged from 0.281 mm to 0.495 mm and were highest along the long axis of tumor 3 and lowest along the long axis of tumor 2. Relative penetration percentage ranged from 3.30% to 10.9% and was highest along the short axis of tumor 3 and lowest along the long axis of tumor 2. The volume fraction of penetrated tumor tissue ranged from 28.04% to 43.42% and was highest for tumor 3 and lowest for tumor 1. A summary of the penetration depths can be found in Table 1(a,b) visualization of the drug concentration profiles can be found in Figure 5.

## Discussion

In this work, we expanded our previously developed three-dimensional CFD model for IPC in tumor nodules (Steuperaert et al., 2017) to include realistic geometries and pressure profiles. We modeled three different tumor nodule geometries of different sizes (288 mm³, 121 mm³, 45 mm³) and extracted spatially varying microvasculature related parameters from DCE-MRI images. Using these parameters, pressure fields were simulated in the tumors and drug transport was studied in all three tumor geometries in the presence of the corresponding pressure field.

In any work relying on DCE-MRI data, a tradeoff has to be made between spatial and temporal resolution (Barnes et al., 2012). We opted for a small temporal resolution to extract an animal-specific AIF. Due to this high temporal resolution and the available MRI hardware, we were limited to a single slice and had to extrapolate the vascular properties estimated from this slice to three-dimensional data. Other works in which mice tumors were studied have used AIF functions from literature to allow for a lower temporal resolution (Zhao et al., 2007; Pishko et al., 2011; Magdoom et al., 2012). The extrapolation of data from 2 D to 3D inevitably leads to inaccuracies in the determination of the interior tumor zones that were determined. In the future, a more realistic estimation of vascular parameters in the tumors could be obtained using multiple slices throughout the tumor during the DCE-MRI and applying the data processing workflow for each slice. Recently, Bhandari et al. (2018) used a 3D DCE-MRI sequence combined with a high temporal resolution in the study of human brain tumors with good results. It is, however, impossible to compensate for physiological and hardware differences by extrapolating scan parameters from one setup to another. We found that, upon fitting the DCE-MRI data to Equation (1), not all pixels could be adequately fit. In some cases, this was due to poor signal quality (too much noise) in these pixels and for the second subset of pixels, located at the edge of the ROI, this was because of overestimations in the initial ROI segmentation, most likely caused by partial volume effects (Ballester et al., 2002). Studying the contrast concentration profiles in the border areas of the initial ROI’s could in the future allow for a better distinction between tumor and surrounding tissue and a more refined ROI.

A final group of pixels that could not be fit by Equation (2) were pixels that exhibited the typical signal shape of necrotic zones. Both tumor 1 and tumor 2 were found to have necrotic cores, with the pixels in the dark regions on Figure 3 exhibiting the typical relation between time and signal intensity for necrotic zones as was also found in (Cho et al., 2009). The necrotic zone in tumor 1 (largest tumor), however, was estimated to be a factor 8 larger than the one in tumor 2.

In tumor 3, two different zones could also be distinguished (Figure 2), but neither of them was necrotic. Pixels from the interior zone of tumor 3 displayed a relation between time and signal intensity that was more close to that of a hypoxic zone with a slower contrast uptake and a prolonged wash-out compared to perfused tumor tissue. Contrast concentration curves of pixels belonging to the outer shell of each of the three tumors followed the typical relation for viable, well-perfused tumor tissue with a sharp peak in contrast concentration and a rapid wash-out due to the abundance of leaky, tortuous microvasculature in this area.

The measured pressures varied between 2066 Pa and 2839 Pa with the largest value observed in tumor 2 and the smallest value in tumor 1. Overall, the lower the *LP50*, the steeper the pressure profile and the higher the maximal interstitial fluid velocity, and therefore the higher the radial outward convective flow will be. Calculated pressures showed a similar trend but were lower with values varying from 1384 Pa to 1525 Pa (largest value for tumor 2, smallest value in tumor 1). Tumor 2, which had the largest percentage of viable, well-perfused tumor tissue, presented with the highest IFP. It is important to note that in the CFD model only IFP is simulated by means of the Starling term. However, the invasively measured pressure yields the total pressure, i.e. the sum of the IFP and solid state (SS) pressure (Boucher & Jain, 1992) with the SS pressure transmitted by the solid and elastic elements of the extracellular matrix and cells, as opposed to fluids (IFP). The difference between the measured and predicted IFP pressure may be a measure for the SS matrix pressure that exists in solid tumors (Stylianopoulos, 2017). Nonetheless, validation of this assumption is mandatory but not trivial, as most invasive pressure measurements will correspond to the total stress rather than one of its components. Deformation tests performed by Nia et al. (2016) estimated the solid stress in solid tumors of colorectal and pancreatic origins to be in the range of 90–1000 Pa. Given this order of magnitude of the SS pressure as an indication, the SS pressure may explain the difference between the simulated IFP and the measured total pressure. Assuming that indeed the SS stress can be calculated as the experimentally measured total stress minus the calculated IFP, the SS pressures in the three tumor geometries equals 682 Pa for tumor 1, 1367 Pa for tumor 2, and 1105 Pa for tumor 3. Due to the differences in extracellular matrix (ECM), cell density and the presence of cancer-associated fibroblasts (CAFs), the tumor tissue permeability is likely to be significantly different from the healthy surrounding tissue permeability. While we previously found that the IFP remained virtually constant when the Darcy permeability of the tissue changed, these changes are very likely to impact the SS pressure. To obtain a tumor-specific estimate of the SS pressure to add to the calculated IFP, an extra step could be added to the workflow in future work where – using a similar workflow as described by Helmlinger et al. (1997) and Stylianopoulos et al. (2012) – the tumor deformation after cutting is used to determine the SS pressure.

The invasively measured total pressures in the tumors were within the range of previously measured pressures in tumors of similar size, location and origin (11 mmHg–40 mmHg with a median of 22 mmHg (Gremonprez et al., 2015) which equals a range of 1497–5333 Pa with a median of 2933 Pa).

In our previous work (Steuperaert et al., 2017), we established a positive relationship between tumor size and maximal IFP. In this work, we found that this relationship does not longer hold in the presence of zones with different vascular properties, while we did observe a positive relationship between the percentage of viable tumor tissue and the maximal IFP. This is of particular interest as a recent work by Bhandari et al. (2018) found no relation between tumor size and IFP. The different IFP values found for different tumor volumes in this work are thought to be due to the maximal IFP being reached in the geometries being lower than the maximal possible IFP. When this maximal pressure value is reached, the net volume flux out of the vasculature is zero and exceeding this pressure value would most likely result in the local collapse of the microvasculature. This maximal IFP value can be found by equaling the filtration rate of plasma fluid per unit volume (JV/V) across the vessel wall as described by Starling’s law to zero.

$$\frac{J_{v}}{V}=\frac{L_{p}S}{V}\left(P_{v}-P_{i}-c\left(\pi_{v}-\pi_{i}\right)\right)$$

This results in the following maximal IFP: P_i, MAX_=P_v_−c(π_v_−π_i_) in which P_v_ is the vascular pressure, c the osmotic reflection coefficient and π_v_ and π_i_ the oncotic pressures in blood and interstitium, respectively. Using the parameters used in this work, this maximal interstitial pressure value equals ± 1530 Pa. The pressure obtained in the tumors described in the work of Bhandari et al., is close to the maximal interstitial pressure. Therefore, it is not expected that IFP will be different between different tumors geometries in the work of Bhandari et al. We also noted that the presence of the 3D necrotic zones resulted in heterogeneous IFP profiles in the tumors. We also noted that the presence of the 3D necrotic zones resulted in heterogeneous IFP profiles in the tumors. These findings are of specific interest as previous works using symmetrical, idealized tumor and necrotic core geometries, found little to no influence of the necrotic core on the IFP profile (Baxter & Jain, 1990). A similar finding was done by Pishko et al. (2011), where skewed IFP values were reported based on IFP calculations using 3D spatially varying tissue parameters.

The absolute penetration depths calculated in this work ranged from 0.32 mm to 0.50 mm. When comparing these results with those obtained in previous studies, they were found to be in good agreement with the experimentally defined range of 0.41–0.56 mm where carboplatin (another platinum-based drug of roughly the same size) was used (Ansaloni et al., 2015). The observed concentration profiles were highly dependent on both the probing location and direction. We calculated the volume percentage of each tumor where the local drug concentration after 30 min of IPC exceeded the *IC50* value of cisplatin (De Vlieghere et al., 2016) and found a range of 28.04–43.42%, with the highest percentage occurring in tumor 3 and the lowest in tumor 2. In our previous work, we explored the relative importance of several different parameters that influenced the drug transport during IPC. One of the largest improvements in penetration depth was obtained by subjecting the tumor nodules to vascular normalization (VN) therapy with the APD doubling in certain cases. The results in this work indicate that penetration depths in certain tumors (i.e. tumor 3) could reach up to 47% when doubled and might be very good candidates for VN therapy.

As in all numerical models, some assumptions were made to make the model implementable. The model may incorporate different zones with different vascular parameters, but the reality is more complex. Using a higher spatial resolution and multiple slices (instead of 1 slice) to obtain DCE-MRI data throughout the tumor should allow for a more realistic distribution of vascular parameters in the tumors, possibly on a voxel per-voxel basis. The estimates for *L_p_S/V* could be further refined by coupling model equations to the compartmental model that would yield a direct estimation of *L_p_S/V* values from the tracer signal instead of the proportionality to *J_v_/V* approximation (Zhao et al., 2007). The model could be further refined by the addition of sink terms that represent the effect of other physiological phenomena, such as drug binding to the ECM and plasma protein binding. Additionally, a number of refinements could be made to the boundary conditions of the model, such as the inclusion of time-dependent boundary concentrations to take into account the changing concentration of drug in the carrier fluid and non-zero boundary pressures to take into account the hydrostatic pressure head of the fluid in the abdomen. An additional sensitivity study using this model may include changing the drug concentration boundary concentration over a biologically relevant range to quantify possible penetration depth enhancements by using higher concentrations.

The results presented in this work are based on data obtained from three tumors in a mouse model. Applying the same workflow, with the possible inclusion of some of the adaptations mentioned above, to a larger group of animals would allow for the determination of a population-based average of the AIF and could also shed more light on the extent of tissue heterogeneity within different tumor geometries of the same origin. Applying the same protocol to human subjects should be feasible but there are a few aspects to take into consideration. Although DCE-MRI sequences for human tumor imaging exist, the location of the PM makes them susceptible to motion artifacts due to both respiration and peristaltic movement which could interfere with data quality. Additionally, the noninvasive pressure estimation presented in this work only estimated IFP pressures and does not account for SS pressures. To obtain an accurate, noninvasive estimation of total tumor pressure, the SS should be estimated noninvasively as well.

In conclusion, we expanded our previously developed 3D CFD model of the drug mass transport in a single tumor nodule during IP chemotherapy to include realistic tumor geometries and spatially varying vascular properties. DCE-MRI studies made it possible to distinguish between tumorous tissues with different vascular properties as well as the healthy surrounding tissues and necrotic zones. We found that tumor size no longer correlated to the maximal IFP when regions with different vascular properties were included. Using realistic geometries of both tumor nodules and necrotic cores had a big impact on the resulting penetration depth in this work, unlike the previous model in which the inclusion or absence of a necrotic core did not seem to influence the penetration depth significantly. We found that the resulting pressure profiles within tumors were highly dependent on the irregular geometries and different zones, indicating a strong need to include both aspects in the model. The total pressure was found to be higher for higher percentages of viable tumor tissue volume ratios. The presence of a significant solid-state pressure in the tumor nodules may explain the difference between calculated IFP pressures and measured total pressures.
